# Understanding and Managing Ménière’s Disease: A Comprehensive Case Report

**DOI:** 10.7759/cureus.103960

**Published:** 2026-02-20

**Authors:** Manal M El Faham, Reham Elrashidy

**Affiliations:** 1 General Medicine, Alexandria National University – Faculty of Medicine, Alexandria, EGY; 2 Audio-Vestibular Medicine, Otolaryngology, Hearing and Balance Clinic, Dubai Hospital, Dubai Academic Health Corporation, Dubai, ARE; 3 Audio-Vestibular Medicine, Mohammed Bin Rashid University of Medicine and Health Sciences, Dubai, ARE

**Keywords:** case report, meniere’s disease, sensorineural hearing loss, vertigo, vitamin d deficiency

## Abstract

Ménière’s disease (MD) is a chronic vestibular disorder characterized by recurrent vertigo, fluctuating sensorineural hearing loss (SNHL), tinnitus, and aural fullness, with an unpredictable course that can significantly impair quality of life. Endolymphatic hydrops (EH) remains the hallmark histopathological finding; however, its underlying pathophysiology is not fully understood and likely reflects a multifactorial process involving immune, viral, allergic, genetic/familial, and vascular mechanisms. Emerging evidence suggests that EH may represent a secondary manifestation rather than the primary driver of symptoms, potentially resulting from endolymphatic sac hyperplasia. Given this complexity, no single unifying mechanism has been confirmed, and etiologic contributors may vary among patients. Presenting a clinical case, therefore, provides valuable insight into this heterogeneity and highlights the need for continued research to clarify the underlying pathology of MD.

This report presents a 57-year-old male with recurrent vertigo, progressive moderately severe right-sided SNHL, tinnitus, and severe vitamin D deficiency. Diagnostic pure-tone audiometry, comprehensive vestibular testing, and imaging were performed. Management consisted of lifestyle and dietary modifications, pharmacologic therapy, vestibular rehabilitation, vitamin D supplementation, and hearing aid rehabilitation, resulting in improved vertigo control, balance, and overall quality of life. The discussion integrates current diagnostic criteria, differential considerations, and evidence-based management strategies, emphasizing individualized, patient-centered care and the importance of recognizing emerging modifiable factors such as vitamin D deficiency and potential genetic susceptibility.

The central aim of this case report is to enhance medical students' understanding of this multifaceted disorder and to underscore the clinical reasoning skills required for accurate and timely diagnosis.

## Introduction

Ménière’s disease (MD) is a disabling vestibular disorder that affects approximately 0.2% of the population, with onset typically between ages 40 and 60 years [1,2]. Although MD shows a female predominance in most epidemiological studies, with reported female-to-male ratios ranging from 1.13:1 to 4.33:1, our patient was male. While less common, male cases are well documented in the literature and remain an important part of the disease spectrum [3]. It is characterized by recurrent episodes of vertigo, fluctuating sensorineural hearing loss (SNHL), tinnitus, and aural fullness [4-6]. The unpredictable nature of the symptoms often leads to significant psychological distress and functional impairment, profoundly diminishing quality of life [1]. Despite decades of research, the precise etiology of MD remains incompletely understood, limiting the development of definitive curative therapies [6]. Consequently, current management focuses primarily on symptom control, hearing preservation, and improvement of daily functioning.

The most widely accepted pathophysiological theory of MD is endolymphatic hydrops, an abnormal distension of the endolymphatic spaces within the membranous labyrinth caused by excessive accumulation of endolymphatic fluid [4,6]. Although endolymphatic hydrops is considered a hallmark histopathological finding consistently demonstrated in postmortem specimens, its underlying pathophysiology remains incompletely understood. Current evidence suggests that the mechanisms driving its development are multifactorial and may include viral infections, autoimmune processes, vascular insufficiency, and genetic predisposition [5,6].

Distension of the membranous labyrinth alters the normal homeostasis of the inner ear by increasing intralabyrinthine pressure, distorting the delicate sensory structures of the cochlea and vestibular apparatus responsible for hearing and balance [4]. Permanent or progressive hearing loss in MD is primarily associated with ruptures of the membranous labyrinth, which result in irreversible sensory and neural damage [4,6]. According to the 2025 hyperplasia-based theory reported in Scientific Reports, based on recent evidence from Bryton et al. (2025), findings suggest that pathological changes in temporal bones with MD are not solely due to hydrostatic distension but reflect hyperplastic growth of the endolymphatic epithelium. This model provides compelling morphological evidence that endolymphatic hydrops may be a secondary manifestation rather than the primary driver of symptoms, consistent with the observation that the classical pressure-based model lacks direct experimental support. In addition, hyperplastic epithelial remodeling may better explain progressive sensory dysfunction and the observed mismatch between hydrops severity and clinical symptoms [7]. These findings represent a major paradigm shift, suggesting that MD may be fundamentally a degenerative epithelial disorder rather than a purely hydrodynamic one, challenging longstanding assumptions about the role of endolymphatic hydrops and reshaping future diagnostic and therapeutic strategies [7]. This perspective also aligns with clinical observations that hydrops severity does not always correlate with symptom intensity and that some patients progress despite stable or mild hydrops. These findings highlight the evolving and heterogeneous nature of MD pathophysiology and underscore the need for continued high-quality research to clarify its underlying mechanisms [6,7]. While rupture-related damage is typically irreversible, our clinical experience aligns with the well-described early course of MD, in which patients often exhibit fluctuating hearing thresholds and episodic vertigo with periods of remission and exacerbation before any permanent deficit occurs. This fluctuation phase is consistent with classical descriptions of the disease’s natural history [8].

**Figure 1:**
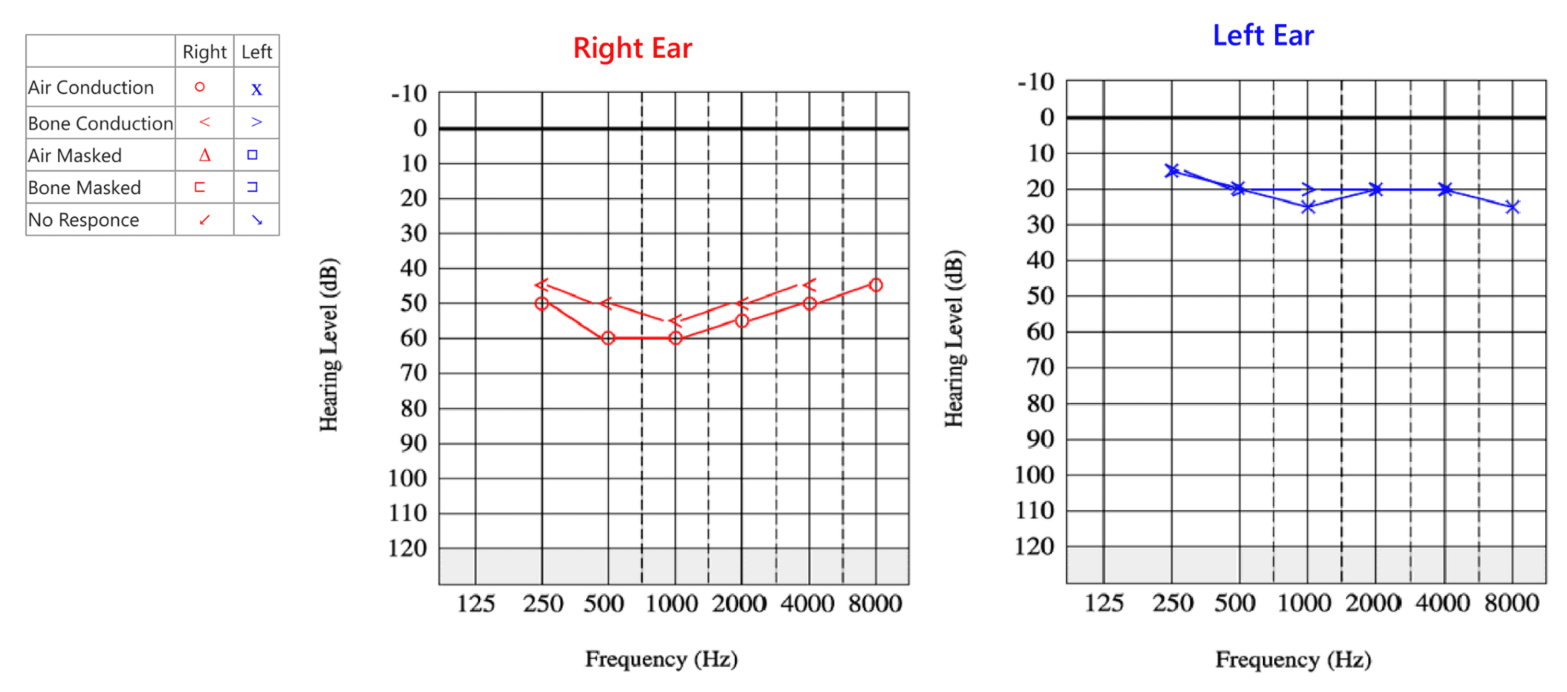
Pure-Tone Audiogram in Chronic Late-Stage Ménière’s Disease (MD). Pure-tone audiogram of the right (red) and left (blue) ears. This single audiogram represents both pre- and post-treatment evaluation, demonstrating moderate-to-severe sensorineural hearing loss (SNHL) with no measurable improvement, consistent with chronic, late-stage MD [15]. MD: Ménière’s disease, SNHL: Sensorineural hearing loss.

Several studies have demonstrated that low serum vitamin D levels are associated with higher recurrence rates of benign paroxysmal positional vertigo (BPPV), and supplementation has been shown to reduce recurrence in deficient patients [9-11]. Emerging evidence also suggests a possible link between vitamin D deficiency and endolymphatic hydrops, with proposed mechanisms involving impaired calcium homeostasis within the inner ear and altered otoconial metabolism [12,13]. Although the exact causal relationship in MD remains under investigation, the growing body of literature supports the role of vitamin D as a modifiable risk factor that may influence disease activity in susceptible individuals, as illustrated in our patient’s pre- and post-treatment audiogram (Figure 1) [12,13].

Accurate diagnosis of MD is critical because multiple vestibular and otologic disorders can mimic its presentation. These include vestibular migraine, BPPV, acute vestibular syndrome, autoimmune inner ear disease (AIED), vestibular schwannoma, perilymph fistula, superior semicircular canal dehiscence (SSCD), otosyphilis, transient ischemic attack (TIA) of the posterior circulation, and multiple sclerosis (MS) [2,5]. A comprehensive evaluation, including a detailed clinical history, audiovestibular testing, and appropriate neuroimaging, is essential to distinguish MD from these conditions [2,5]. Early recognition, differentiation from other vestibular disorders, and accurate classification of disease as definite or probable based on established consensus criteria remain essential for guiding appropriate management and improving patient outcomes [2].

Given the heterogeneity of MD and the absence of a single definitive pathophysiological mechanism [4-8,12-14], a clinical case provides a practical framework to illustrate disease presentation, diagnostic reasoning, and real-world management, reflecting the clinician’s role in managing symptom progression despite underlying etiologic uncertainty.

## Case presentation

A 57-year-old male, previously residing in the United Arab Emirates and currently living in Egypt, presented with a long-standing history of intermittent, recurrent episodes of vertigo, tinnitus, and fluctuating right-sided hearing loss. The patient, a retired pharmaceutical sales manager, was otherwise healthy and a non-smoker, with no history of chronic systemic illness or prior otologic surgery. He first experienced true spinning vertigo in his mid-30s, with episodes lasting approximately 20 minutes and often preceded by right-sided tinnitus, a sensation of aural fullness, nausea, and occasional vomiting. Initially, attacks occurred two to three times per month, each followed by hours of imbalance and fatigue. Over time, both the frequency and intensity of vertigo episodes increased. Between episodes, he remained relatively symptom-free except for transient imbalance with rapid head or body movements. He reported no headache, photophobia, or phonophobia to suggest vestibular migraine.

His family history was notable for vertigo in his mother and one sister, suggesting a possible familial predisposition. Other familial conditions included paternal coronary heart disease and maternal breast cancer, with no additional inherited or metabolic disorders reported. The patient denied prior exposure to ototoxic medications and was not on any long-term therapy before symptom onset.

On interictal general examination, vital signs were stable, and no systemic abnormalities were noted. Otoscopic examination revealed intact tympanic membranes bilaterally without evidence of middle-ear pathology. Neurological evaluation, including cranial nerves, was unremarkable.

The patient first presented during an acute episode of rotational vertigo consistent with a right-sided MD attack. During the symptomatic phase, bedside examination demonstrated horizontal-torsional nystagmus beating away from the affected (right) ear, supporting a right-sided peripheral vestibular origin.

Bedside vestibular testing was repeated during the interictal period, approximately two to five days after the acute attack, when the patient was free of vertigo. At that time, no spontaneous nystagmus was observed, and oculomotor testing, including smooth pursuit, saccades, and gaze-holding, was normal. Postural assessment revealed mild instability on the Sharpened Romberg test and a rightward deviation on the Fukuda stepping test. The bedside Head Impulse Test (HIT) elicited overt corrective saccades with rightward head rotation, indicating a right-sided unilateral vestibular weakness. The Dix-Hallpike maneuver and positional testing were negative, effectively ruling out BPPV. Coordination testing was normal. Collectively, these findings suggested an uncompensated right-sided peripheral vestibular dysfunction.

Audiologic evaluation performed during a stable interictal period demonstrated moderate-to-severe SNHL in the right ear (Figure 1), consistent with chronic Stage III MD. Speech discrimination was 72% on the right and 100% on the left. Tympanometry showed Type A curves bilaterally, and acoustic reflexes were absent on the right side, indicating normal middle-ear function.

A repeat bedside vestibular examination several days later again showed no spontaneous nystagmus, with findings otherwise unchanged. Formal vestibular laboratory testing was initially deferred because of poor patient tolerance.

When tolerated, vestibular laboratory assessment was completed and included video head-impulse testing (vHIT), caloric irrigation, and cervical vestibular-evoked myogenic potentials (cVEMP). Results demonstrated a reduced right-sided vHIT gain of approximately 0.6 with overt corrective saccades, a unilateral caloric weakness exceeding 28%, and an absent or markedly diminished right-sided cVEMP response. This constellation of abnormalities across high-frequency (vHIT), low-frequency (caloric), and otolith (cVEMP) pathways indicated moderate-to-severe unilateral right-sided peripheral vestibular hypofunction. Although caloric-vHIT dissociation is frequently reported in MD, the presence of vHIT abnormalities in this case likely reflects chronic peripheral vestibular involvement with incomplete central compensation, particularly because testing was performed during the interictal period rather than during an acute attack.

Despite the long disease duration, nearly two decades, the patient experienced an increase in vertigo frequency, an atypical but documented late-phase pattern in MD. Left-sided vestibular function remained normal across all modalities, with no evidence of contralateral involvement at this stage.

Routine laboratory investigations revealed severe vitamin D deficiency (25-hydroxyvitamin D: 12 ng/mL). Complete blood count, fasting glucose, electrolytes, renal and liver function tests, thyroid profile, serum IgE, and autoimmune and infectious markers (antinuclear antibody (ANA), erythrocyte sedimentation rate (ESR), C-reactive protein (CRP), Venereal Disease Research Laboratory test (VDRL)) were within normal limits. MRI of the internal auditory canals demonstrated normal seventh and eighth cranial nerve complexes without mass lesions or demyelination. A bilateral Type I vascular loop was noted but considered clinically insignificant.

Functional impact was quantified using the Dizziness Handicap Inventory (DHI) and Tinnitus Handicap Inventory (THI). At peak symptom severity, the DHI score was 58 and the THI score was 56, both indicating moderate handicap. After three months of medical management and stabilization, the DHI improved to 22 and the THI to 20, reflecting a transition to mild handicap and correlating with the patient’s reported clinical improvement.

Based on clinical, audiologic, vestibular, and radiologic findings, the patient met the 2015 Bárány Society and American Academy of Otolaryngology-Head and Neck Surgery (AAO-HNS) criteria for definite MD [2], with alternative vestibular disorders excluded.

A structured management plan was initiated following diagnostic confirmation, with the specific therapeutic components outlined in the Management section of this report. A follow-up audiogram obtained three months after treatment initiation demonstrated stable, moderately severe SNHL in the right ear, with no additional deterioration. This pattern was consistent with cochlear stabilization or the establishment of permanent hearing loss characteristic of the later stages of MD (Figure 1). Over the same interval, the patient reported a marked reduction in both the severity and frequency of vertigo episodes.

Although the post-treatment audiogram was performed at the three-month mark, the patient continued medical therapy and lifestyle interventions with regular follow-up. Functional improvement was objectively documented: the DHI score improved from 58 to 22, and the THI score improved from 56 to 20, indicating a shift from moderate to mild handicap and aligning with the patient’s subjective clinical progress. The patient also demonstrated improved recognition of symptom triggers, consistent adherence to lifestyle recommendations, and increased satisfaction with hearing rehabilitation strategies.

Management was delivered as a multimodal, individualized approach tailored to the patient’s disease stage, symptom severity, and overall clinical stability. Foundational counseling focused on lifestyle and dietary modifications, including sodium restriction, avoidance of caffeine and other potential triggers, stress reduction, adequate sleep hygiene, and avoidance of ototoxic medications. These measures were emphasized as essential components of long-term disease control.

During acute vertigo episodes, the patient received vestibular sedatives and antiemetics for symptomatic relief. Long-term medical therapy included betahistine and hydrochlorothiazide to reduce vertigo frequency and mitigate endolymphatic pressure fluctuations. These treatments were implemented as part of a coordinated, multimodal strategy rather than as isolated interventions, reflecting the fluctuating and episodic nature of MD.

Vitamin D supplementation was initiated at 2,000 IU twice daily and subsequently tapered to 1,000 IU daily once serum levels normalized. Serial monitoring demonstrated correction of the initial deficiency, with levels rising from 12 ng/mL at baseline to 34 ng/mL and thereafter maintained between 32 and 36 ng/mL. This biochemical improvement corresponded with a reduction in vertigo frequency and enhanced postural stability.

Vestibular rehabilitation therapy (VRT) was introduced to address persistent imbalance between vertigo attacks; however, the patient demonstrated limited tolerance and adherence. A right-sided hearing aid was fitted to address the SNHL and support tinnitus retraining therapy, resulting in improved communication and overall quality of life.

These interventions functioned synergistically, each targeting a distinct aspect of the disease process. Because therapeutic benefit in MD is cumulative rather than attributable to a single modality, isolating the effect of any individual intervention is neither clinically feasible nor expected.

After three months of comprehensive, patient-centered management, the patient experienced a marked reduction in vertigo frequency and severity, improved balance, and enhanced overall quality of life while continuing the established treatment plan. This clinical improvement was supported by objective functional measures: the DHI score improved from 58 to 22, and the THI score improved from 56 to 20, reflecting a shift from moderate to mild handicap and aligning with the patient’s subjective progress.

**Figure 2:**
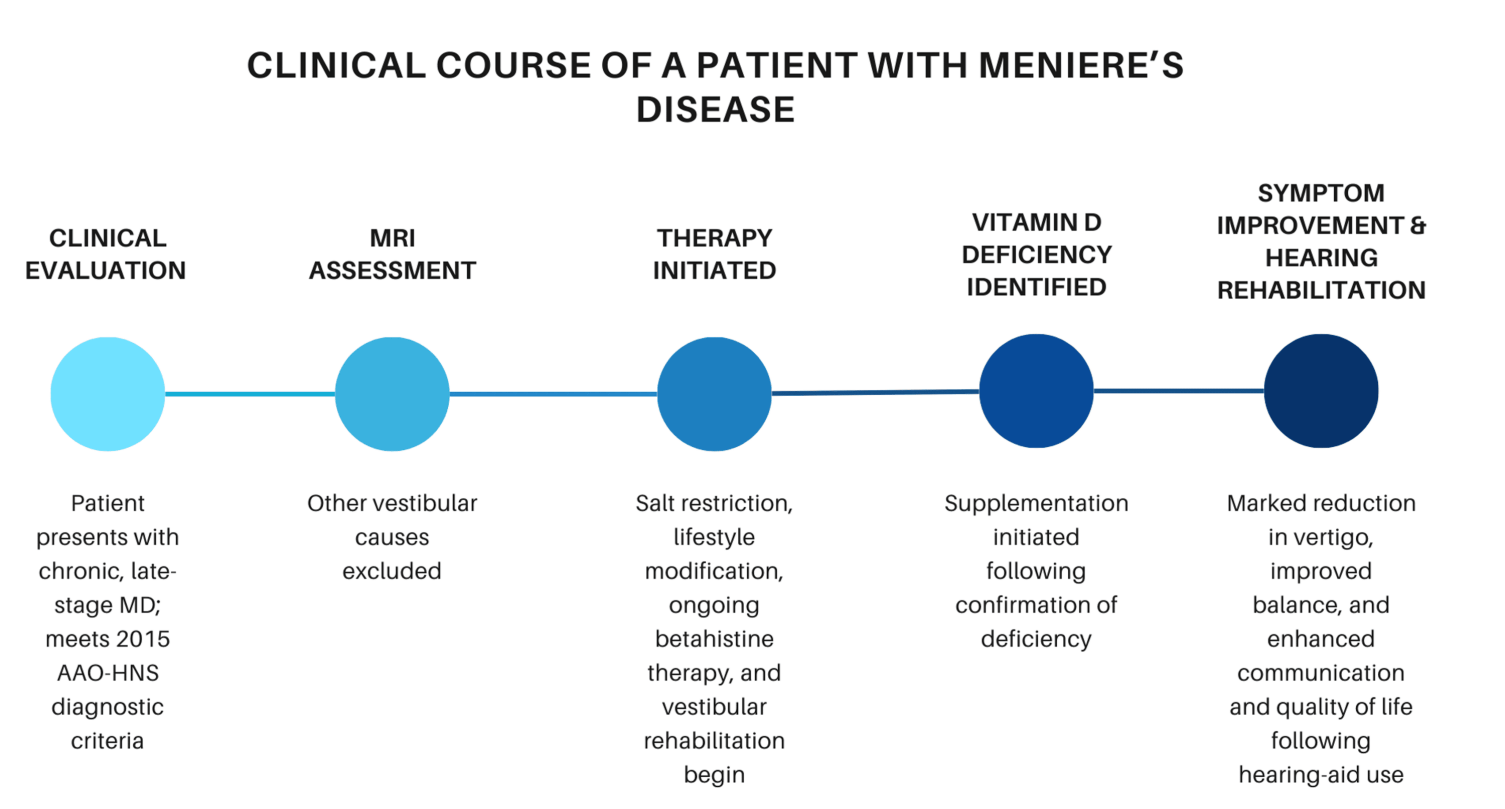
Clinical Course of a Patient With Ménière’s Disease (MD). Timeline of the patient’s clinical course following presentation with chronic, late-stage MD. Key milestones include diagnostic evaluation, initiation of medical and lifestyle interventions, correction of vitamin D deficiency, and subsequent improvement in vertigo, balance, and hearing-related quality of life. MD: Ménière’s disease, MRI: magnetic resonance imaging, AAO-HNS: American Academy of Otolaryngology–Head and Neck Surgery.

Symptom onset and disease progression extended over nearly two decades, and the patient was evaluated during what corresponds to the third stage of the disease, as illustrated in Figure 2 [15].

Our intention in presenting this case is to illustrate this holistic, stepwise, and complementary approach, which mirrors how MD is managed in general practice and across specialties. Given the disease’s variability and episodic nature, it is essential for physicians, including general practitioners, ENT specialists, audiology and vestibular medicine specialists, and neurologists, to recognize the classic presentation and understand the rationale for a multidisciplinary, multimodal management plan.

## Discussion

MD is a chronic, fluctuating inner-ear disorder with a multifactorial etiology. Proposed contributing mechanisms include autoimmune processes, viral infections, vascular insufficiency, and genetic susceptibility, all of which may influence endolymphatic homeostasis and disease expression [5,14]. Recent experimental and histopathological studies suggest that epithelial hyperplasia within the endolymphatic sac may contribute to endolymph accumulation and the manifestation of vestibular symptoms [7,8]. Emerging evidence also highlights the potential role of vitamin D deficiency in vestibular disorders. Vitamin D is essential for calcium regulation and otoconial metabolism, and deficiency may impair otolith stability, increase susceptibility to vestibular dysfunction, and exacerbate vertigo recurrence [9-13]. In this case, the patient presented with severe vitamin D deficiency, and correction of serum levels coincided with improved vertigo control and postural stability, underscoring its relevance as a modifiable risk factor in MD.

Management of MD remains individualized and multimodal, reflecting the heterogeneity of disease presentation and progression. First-line strategies emphasize lifestyle and dietary modifications, including salt restriction, adequate hydration, stress reduction, and avoidance of caffeine and ototoxic medications [1]. Pharmacologic therapy commonly includes betahistine and diuretics such as hydrochlorothiazide, which aim to reduce endolymphatic pressure and stabilize vestibular function. For patients with persistent or refractory symptoms, intratympanic steroid injections or gentamicin therapy may be considered. Surgical options, including endolymphatic sac decompression or, in advanced cases, labyrinthectomy, are reserved for severe, treatment-resistant disease.

VRT is not typically recommended during the active, fluctuating phase of MD because of limited evidence of benefit and the unpredictable nature of vestibular function during this period. Instead, VRT is more effective in later stages or following ablative interventions, when vestibular compensation can occur more consistently [16-20]. In this case, VRT was attempted but not well tolerated. Hearing rehabilitation, including the use of a right-sided hearing aid, contributed to improved communication and supported tinnitus retraining therapy, reinforcing the importance of multidisciplinary care.

Regular follow-up is essential in MD to monitor hearing thresholds, vertigo frequency, and treatment response [1]. Ongoing evaluation allows clinicians to adjust therapy, implement rehabilitative strategies, and address modifiable contributors such as vitamin D deficiency. Recognition of emerging risk factors, including nutritional deficiencies and familial predisposition [9-14], may refine prognostic assessment and guide personalized management. Future research should continue to explore molecular and genetic pathways involved in MD, with the goal of identifying predictive biomarkers and developing targeted, disease-modifying interventions.

## Conclusions

MD is a complex, chronic vestibular disorder marked by episodic vertigo, fluctuating SNHL, tinnitus, and aural fullness. Its multifactorial etiology and overlapping clinical presentations continue to challenge timely diagnosis and effective management. This case underscores the value of a patient-centered, stepwise approach that integrates clinical assessment with metabolic evaluation and comprehensive audiovestibular testing. Early recognition using established consensus diagnostic criteria remains essential for distinguishing MD from other vestibular conditions and initiating appropriate, individualized care.

Optimal management requires a combination of lifestyle and dietary modifications, pharmacologic therapy, and structured monitoring. Attention to emerging modifiable contributors, such as vitamin D deficiency, may further influence symptom severity, recurrence, and long-term stability. A multidisciplinary strategy, including hearing rehabilitation and vestibular training when appropriate, plays a critical role in improving functional outcomes and overall quality of life.

Consideration of precision medicine factors, including familial or genetic predisposition, highlights the need for tailored therapeutic planning. Sustained follow-up, patient education, and identification of individual triggers enhance symptom control, reduce relapse, and empower patients to participate actively in their care. Continued research into molecular, genetic, and metabolic pathways may lead to future disease-modifying therapies and refine personalized management strategies for MD.
